# Stability of fruit ripening traits of banana (*Musa species*) across postharvest environments

**DOI:** 10.1016/j.heliyon.2024.e37143

**Published:** 2024-08-30

**Authors:** Muluken Bantayehu, Melkamu Alemayehu

**Affiliations:** Department of Plant Sciences, College of Agriculture and Environmental Sciences, Bahir Dar University, Bahir Dar, Ethiopia

**Keywords:** Mechanical damage, Grand naine, AMMI, GGE, Biplot

## Abstract

Banana (*Musa species*) is the fourth most important export crop worldwide after cereals, oil crops and sugar. In spite of this socio-economic significance, the crop suffers massive postharvest losses caused by mechanical fruit damage, limited infrastructure for fruit ripening, postharvest diseases and physiological disorders. Although use of optimum postharvest environments such as packaging and storage temperatures can reduce fruit loss and improve ripening quality; information regarding the interaction between varieties and postharvest environments and stability of fruit ripening traits across postharvest environments is limited. The objectives of this study were to determine the magnitude of interaction of varieties with postharvest environments on fruit ripening traits and to identify stable banana variety for ripening across postharvest environments. Seven commonly grown banana varieties (Dwarf Cavendish, William I, Grand Naine, Poyu, Giant Cavendish, Butazu and Local variety) were laid out in a completely randomized design with five replications in ten varied postharvest environments. The result indicated that pulp and peel ratio had negative high principal component one (PCA1) score whereas the PCA1 score for postharvest period, peel weight and fruit weight were positive and high. Cluster analysis grouped Dwarf Cavendish and Grand Naine; Poyu and Butazu varieties together for postharvest traits whereas the local variety was clustered separately. This study has demonstrated that hybridization of local with the introduced varieties can be done to improve postharvest traits. AMMI depicted significant variation for genotype, postharvest environments and their interactions for all traits. The magnitude of environmental effect was higher than the genotype and interaction effects. AMMI and GGE biplot analyses identified Gran Naine, Poyu and William I as consistent for ripening traits across postharvest environments.

## 1 Introduction

Banana is the fourth most important crop in world export trade after cereals, oil crops and sugar with annual global production of 124 million metric tons in 2021 [1]. However, banana fruit postharvest losses, especially in Africa are incurred at early stages of the food value chain, mainly due to poor handling during harvesting, storage and cooling facilities [2]. FAO estimated that postharvest losses can reach up to 40 percent of the overall banana fruit yield in developing countries [3]. The estimated postharvest loss of banana in the traditional marketing system ranged from 20 to 80 %, caused by mechanical damage and desiccation [4].

Mechanical damage is a major factor that leads to downgrading of banana fruits in the world market. Quality rating in the European Union takes into account the percentage of peel damage due to bruising, scarring and scratching [5]. Peel weight offers advantage in protecting banana fruits against mechanical damage during transport, handling and shipping. Banana varieties with high peel weight are often more suitable for international banana trade, as the peel helps the fruit to resist mechanical damage during transportation [5].

Although in recent times, modern orchards started producing newly introduced high yielding varieties such as Dwarf Cavendish, William I, Grand Naine, Poyu, Giant Cavendish and Butazu in Africa; information about variability and stability of fruit ripening traits of these genotypes across diverse postharvest environments is unavailable. Postharvest traits are affected by postharvest environments, related to storage temperature and packaging, which result in differential expression of fruit ripening genes in varied postharvest environments [6]. The phenotypic value of a trait is affected by genotype, environment and genotype by environment interaction. The G × E interaction arises when there is differential response of genotypes to environmental changes [7]. Many studies have been done to understand the effect of G × E interaction on pre-harvest traits; however, research on the effect of genotypes and postharvest environments on ripening traits is scanty in the literature. Identification of stable varieties in ripening traits could be one of the strategies to reduce postharvest losses across varied postharvest environments. The objectives of this study were to determine the magnitude of interaction of variety and postharvest environments and to identify stable banana varieties for fruit ripening traits across postharvest environments.

## 2 Methods

### 2.1 Experimental setup and sampling

Fruit samples of seven commonly grown banana varieties (Dwarf Cavendish, William I, Grand Naine, Poyu, Giant Cavendish, Butazu, and Local variety) were harvested at green maturity stage from Weramit Horticultural Research Station, Adet Agricultural Research Centre, Bahir Dar, Ethiopia, and delivered carefully to the Plant Analysis Laboratory of the Department of Botany, College of Science, Bahir Dar University, Ethiopia. Banana bunches were immediately de-handed and fingers carefully removed and washed with tap water. Surface water on the fingers was allowed to air dry. Only uniform sized fingers were used for this experiment; hence, banana hands from top and bottom of each bunch were discarded.

Fruit fingers taken from each variety were divided into ten groups, with each group containing about thirty fingers. From the ten groups; five groups were put inside perforated polyethylene bags; while the remaining five groups were kept without polyethylene bags. Eight groups of fingers from each variety (four groups packed in perforated polyethylene bags and the other four groups without polyethylene bags) were put on the shelves of four different incubators having the temperatures of 30 °C, 25 °C, 20 °C and 15 °C and constant relative humidity of 90 %. The remaining two groups of fingers (with perforated and without polyethylene packaging) were put at ambient room temperature as control. In the latter case, the fingers were subjected to the maximum and minimum temperatures of 32 °C and 14 °C, respectively. Each group of fingers was randomly assigned to each treatment. The treatments were repeated three times.

### 2.2 Data collection

Data collected include the following.

(a) Postharvest life [(PHP); (days)], it is the number of days from the date of harvesting up to the date at which 30 % of the sample fruits were at senescence stage or color stage 7**;** color stage of banana was evaluated using the methods described by Savarese (2015), where 1 = all green; 2 = green with trace of yellow; 3 = more green than yellow; 4 = more yellow than green; 5 = yellow with green tips; 6 = all yellow; and 7 = all yellow with brown flecks
(b) Weight loss [(WLR); (%)], the weight of fruits measured before the onset and at the end of the experiment; using a sensitive balance and the difference between the initial and final weight expressed as weight loss percent of the initial fruit weight;
(c) Fruit length [(FL); (cm)], the lengths of five randomly selected fruits from each group measured from the base to the tip, using a ruler at ripening; and the mean values computed and used for analysis;
(d) Pulp diameter [(PD); (cm)], was taken by measuring the diameter of five randomly selected fruit samples at the centre, both on the short and long sides, using a Vanier caliper; and the mean values computed and used for data analysis;
(e) Pulp-peel ratio[(PPR); (cm)], was determined from five randomly selected banana fruits from each group, fruits were peeled at the time of ripening and the peels and pulps of the fruits were weighed separately. The pulp weights were divided into the weights of the respective peels and the mean values computed and used for data analysis;
(f) Fruit volume [(FV); ((cm^3^)], the volume of five randomly selected banana fruits at ripening were measured by water displacement method
(g) Fruit weight [(FW); (g)], the weights of five randomly selected banana fruits were weighed and the mean values computed and used for analysis.

### 2.3 Data analysis

SAS, Genestat and Past 3 softwares were employed for data analysis.

**Multivariate analysis:** Principal component and cluster analysis were used to detect relationship between postharvest traits and banana varieties;

**Stability analysis:** Bartlett′s test for homogeneity of variances was carried out to determine the validity of the individual and combined analyses of variance. Thereafter, AMMI analysis of variance, summarizing most of the magnitude of genotype × environment interaction into one or few interaction principal component analysis, was performed [8,9]. The larger the IPCA scores, either negative or positive, the more specifically adapted a genotype is presumed to a certain environments. The smaller the IPCA scores, the more stable the genotype is over all environments studied.

GGE biplot analysis was also done; while the methods of Finlay and Wilkinson’s [10] and Eberhart and Russell [11] were applied to calculate the regression coefficient (bi), and deviation from regression (Sdi2). It was calculated by regressing mean value of individual genotypes on environmental index. Shukula stability variance (iσ2) [12], cultivar superiority measure and Wricke Ecovalence (Wi_2_) [13] were also computed, where minimum values are considered stable.

## 3 Results

i) Association of Ripening Traits and Banana Varieties

The distribution of fruit traits of banana varieties across postharvest environments are presented in Figure one. The introduced banana varieties had greater fruit weight, peel weight and longer postharvest periods than the local variety. Based on principal component analysis, a positive and high PCA 1 was found for peel weight, fruit weight, fruit length and fruit weight; however, it was negative and high for pulp peel ratio and pulp diameter (Fig. 2).

The biplot of the PCA placed fruit length, fruit weight, peel weight and postharvest period as well as Grand naine and Dwarf cavendish near each other; whereas, peel diameter, pulp peel ratio and weight loss ratio as well Butazu and William I, scattered near each other (Fig. 2). Poyu variety, with small PCA1 and 2 were kept near the origin of the biplot. The local variety was placed separately from the other varieties suggesting different postharvest characteristics. Based on the cluster analysis, Grand Naine and Dwarf cavendish; Poyu and Butazu clustered together; whereas the local variety clustered uniquely (Fig. 3).

The correlation analysis depicted that postharvest period was highly and positively correlated with peel weight, fruit weight, and fruit length; however, it was negatively correlated with pulp peel ratio and pulp diameter (Table 1). Weight losses were significantly and positively correlated with fruit weight and fruit volume.

ii) Stability of fruit ripening traits across postharvest environments

**Table 1:** Simple linear correlation of fruit quality traits of seven banana varieties grown in Ethiopia.

	WLR	FL	FW	PW	PPR	PD	FV
PHP	0.05^ns^	0.42**	0.52**	0.61**	−0.71**	−0.45**	0.37
WLR		0.31	0.53**	0.31	−0.05 ^ns^	0.13	0.46**
FL			0.90**	0.91**	−0.68**	−0.30	0.91**
FW				0.92**	−0.62**	−0.26	0.92**
PW					−0.86**	−0.55**	0.87**
PPR						0.82**	−0.56**
PD							−0.10 ^ns^

PHP = Postharvest life; WLR =Weight loss ratio; FL =Fruit length; PD =Pulp diameter; PPR =Pulp-peel ratio; FV =Fruit volume; FW =Fruit weight; PL =Peel weight; *significant difference; ** highly significant (p < 0.05) l; ns = not significant at 5 % probability level.

The combined analysis of variance revealed a highly significant (p < 0.001) variation for the main effect of variety, temperature, packaging, and their second and third order interaction for all postharvest traits (Table 2). This indicates that genotypes, environments and their interaction were important in governing the expression of these traits across postharvest environments.

**Table 2:** Combined analysis of variance of the seven banana varieties with respect to packaging at different temperatures.

Source of Variation	Mean Squares					
	PL	FL	FW	FV	PPR	PD
Var	2452.68**	214.10**	13685.31**	11331.35**	8.56**	0.99**
Temp	1534.81**	18.00**	4628.18**	4680.00**	6.54**	0.12**
Pack	12923.77**	45.32**	26958.49**	33388.98**	46.80**	1.12**
Var × Temp	105.15**	9.17**	410.71**	423.99**	0.57**	0.10**
Var × Pack	407.56**	9.03**	981.27**	1372.41**	0.86**	0.16**
Temp × Pack	51.44**	1.66**	510.50**	179.01**	0.54**	0.15**
Var × Temp*Pack	61.74**	3.32**	377.51**	458.53**	0.34**	0.10**
Error	15.67	1.17	119.84	86.07	0.08	0.01

FL =Fruit length; PD =Pulp diameter; PPR =Pulp-peel ratio; FV = Fruit volume; FW =Fruit weight; PL = Peel weight; *significant difference at 5 % probability level; **highly significant.

1 % probability level.

The presence of variation between packaging at different temperatures revealed the presence of dissimilarity between the various postharvest environments. The genotypic effect depicted clear variation among banana varieties; therefore, identification of better varieties is crucial for reducing postharvest loss in quality and quantity during postharvest period. The significant variety and temperature, variety and packing, variety packing and temperature interactions indicated the differential varietal performance across postharvest environments. It reduces the association between phenotypic and genotypic values, and thus, genotypes that perform well in one environment perform poorly in another [14].

a) AMMI Analysis of Variance

AMMI analysis of variance depicted significant difference for genotype (G), environment (E), and GxE interaction for peel weight, pulp peel ratio and fruit weight (Table 3). Accordingly, 42.62 % of the total sum of squares (SS) of pulp peel ratio was attributed to environmental effects while 29.20 and 15.28 % were attributed to genotype and GxE interaction, respectively. In case of peel weight, 42.99, 32.83 and 14.39 % of the total sum of squares (SS) were attributed to environmental effects, genotype and GxE interaction, respectively. About 13.2, 25.28 and 43.68 % of the sum of squares were because of genotype by environment, environment and genotype, respectively, for fruit weight. Effect of genotype was high in controlling fruit weight as compared to environment and genotype by environment. Results from analysis of multiplicative effects for pulp peel ratio also showed that the first interaction principal component axis (IPCA 1) captured 57.91 % of the interaction SS. Similarly, the IPCA 2 explained a further 18.82 % of the GxE interaction sum of squares. Results from analysis of multiplicative effects for peel weight also showed that the first interaction principal component axis (IPCA 1) captured 45.12 % of the interaction SS. Similarly, the IPCA2 explained further 28.38 % of the GxE interaction SS, respectively. F-test at P = 0.01 revealed that the first two principal component axes of the interaction were significant for the model. Results from analysis of GxE interaction effects for fruit weight also showed that the first interaction principal component axis (IPCA 1) captured 37.4 % of the interaction SS. Similarly, the IPCA 2 farther explained 33.23 % of the GxE interaction SS, respectively. F-test at P = 0.01 revealed that the first two principal component axes of the interaction were significant for the model. The prediction assessment indicated that AMMI 2 with only two interaction principal component axes was the best predictive model [9]. Further interaction principal component axes captured mostly noise and therefore did not help to predict validation observations. In total, the AMMI 2 model (G + E + IPCA 1 and IPCA 2) contained much of the total SS for pulp peel ratio, peel and fruit weight respectively, indicating that, the AMMI model fits the data well, and validates the use of AMMI 2.

**Table 3:** AMMI analysis of variance of seven banana varieties across ten postharvest environments (packaging and temperatures).

Source of variation	DF	PPR			PL			FW		
		SS	MS	%SS	SS	MS	%SS	SS	MS	%SS
Total	349	176.26	0.51		44822	128.4		187986	539	
Treatments	69	153.54	2.23	87.11	40436	586	90.21	154430	2238	82.15
Genotypes	6	51.47	8.58**	29.2	14716	2452.7**	32.83	82112	13685**	43.68
Environments	9	75.13	8.35**	42.62	19269	2141**	42.99	47513	5279**	25.28
Rep (Env)	40	3.51	0.09	1.99	620	15.5	1.38	4449	111	2.4
Interactions	54	26.94	0.5**	15.28	6451	119.5**	14.39	24805	459**	13.2
IPCA 1	14	15.6	1.12**	57.91	2911	207.9**	45.12	9278	663**	37.4
IPCA 2	12	5.07	0.42**	18.82	1831	152.6**	28.38	8243	687**	33.23
Residuals	28	6.26	0.22	23.24	1709	61	26.49	7284	260	29.4
Error	240	19.21	0.08	10.9	3767	15.7	8.4	29107	121	15.48

DF = degree of freedom; SS = Sum of squares; MS = Mean of square; %SS = percent sum of square; PPR = Pulp-peel ratio; PL = Peel weight; FW = Fruit weight; *significant difference at 5 % probability level; **highly significant 1 % probability level.

Interaction of the seven banana varieties across ten postharvest environments were predicted by the first two principal components of genotypes scores (Table 4). The IPCA scores of a genotype provide indicators of the stability of a genotype across environments [15]. Regardless of the positive or negative signs, genotypes with large scores have high interactions (unstable), whereas genotypes with small IPCA scores close to zero have small interactions and are stable [9]. The lowest IPCA1score was for G3, G6, G5 and G1, G6, and G4 scored low IPCA2 value for pulp peel ratio and these varieties exhibited consistent ripening across postharvest environments. G2, G7 and G5 had small IPCA1 and G4, G2 and G3 scored low IPCA2 value for peel weight on the other hand fruit weight of G3, G6, were low for IPCA1 value and IPCA2 value of G1, G4 and G5 were small indicating stability of these genotypes in varied postharvest environments(Table 4).

b) AMMI and GGE Biplots

**Table 4:** Mean, IPCA1 and IPCA2 values of ripening traits of seven banana varieties evaluated across ten postharvest environments.

		PPR			PW			FW	
Varieties	Mean	IPCA1	IPCA2	Mean	IPCA1	IPCA2	Mean	IPCA1	IPCA2
G1	3.17	−1.19	−0.01	15.61	3.22	1.44	630.58	2.34	−0.94
G2	2.05	−1.1	0.05	33.7	−1.89	1.09	100.44	−3.45	3.81
G3	2.31	0.21	0.56	37.07	−2.93	1.24	114.69	0.12	2.07
G4	2.18	0.33	0.13	33.19	1.01	0.21	102.14	2.06	−0.87
G5	2.24	0.27	0.19	29.82	0.67	1.12	94.23	3.12	1.75
G6	1.93	0.24	−0.13	33.7	0.25	−2.66	92.56	−0.87	−3.21
G7	2.56	0.25	−0.79	31.8	−0.32	−2.44	109.11	−3.31	−2.61

PPR = Pulp-peel ratio; PL = Peel weight; FW = Fruit weight; G1 = Local Variety; G2 = Dwarf Cavandish; G3 = William I; G4 = Grand Naine; G5 = Poyu; G6 = Giant Cavandish; G7 = Butazu.

Biplot analysis displays the two-way data and allows visualization of the interrelationship among environments, genotypes, and interactions between genotypes and environments. Two types of biplots, the AMMI biplot [16,8] and the GGE biplot [17,18] have been used widely to visualize genotype × environment interaction. GGE biplot and AMMI model explained 85.98 % and 76.77 % of the sum of square for peel pulp ratio; 86.57 % and 73.51 % for peel weight; 89.62 % and 70.63 % for fruit weight, respectively (Table 5). The result indicated that GGE explain much of the variation as compared to AMMI model. According to AMMI2, G6, G4 and G5 were the most stable genotype with high pulp peel ratio (Fig. 4a); G2, G4 and G5 were the most stable genotype with high peel weight (Fig. 4c); G1, G3, G4 was the most stable genotype with high fruit weight (Fig. 4e). On the other hand, GGE biplot revealed that G3, G4 and G5 as stable across postharvest environments for pulp peel ratio (Fig, 4 b); G2, G3 and G4 as stable across postharvest environments for peel weight (Fig. 4d); and G2, G3 and G4 as stable across postharvest environments for fruit weight (Fig. 4f). G4, G2, G5 and G3 are the most stable varieties considering these traits across postharvest environments. Therefore, Gand naine, William I and Poyu have stable postharvest qualities suitable for varied postharvest environments. Even if Dwarf Cavendish has stable postharvest quality, its short height might make it less preferable for production by producers.

c)Parametric stability analysis for genotypes

**Table 5:** Trait wise principal component 1 and 2 variance (PCA1 and PCA2) of total GE of seven banana varieties across ten postharvest environments.

Trait		GGE			AMMI	
	PCA1	PCA2	Sum	PCA1	PCA2	Sum
PPR	72.27	13.71	85.98	57.93	18.84	76.77
PW	74.52	12.06	86.58	45.12	28.39	73.51
FW	78.89	10.73	89.62	37.40	33.23	70.63

PPR-Pulp-peel ratio; PL-Peel weight; FW-Fruit weight.

The consistency of fruit ripening traits of banana varieties across postharvest environments using Finlay and Wilkinson’s regression coefficient, Eberhart and Russell’s sum of squared deviations from regression, Wricke ecovalence and Shukula stability variance were performed for peel weight, pulp peel ratio and fruit weight. According to Finlay and Wilkinson’s regression coefficient, Eberhart and Russell’s sum of squared deviations from regression, genotype G2, G6 and G3 were the most stable in peel pulp ratio; genotype G4 and G5 for peel weight; genotype G4 and G2 for fruit weight because it had high trait value, its regression coefficient was almost near to unity and the least deviation from regression (Table 6, Table 7, Table 8). The result of Wricke’s ecovalence and Shukula’s stability variance showed that G2, G4 and G5 were comparatively stable for pulp peel ratio; G4 and G5 were comparatively stable for peel weight; G3, G4 and G5 were comparatively stable for fruit weight as their contribution to the GxE interaction sum of squares was least and with minimum stability variance (σ_i_^2^). Similar results were found by cultivar superiority measures (Table 6, Table 7, Table 8). Genotypes G3, G4 and G5 were identified to show small change in fruit ripening traits across postharvest environments.

**Table 6:** Parameteric stability analysis of varieties for pulp peel ratio across postharvest environments.

Variety	Mean	Wricke ecovalence	Stability variance	Cultivar Superiority	Bi	SDi
Local Variety	3.170	2.5096	0.5315	0.0040	1.0330	0.3389
Dwarf Cavandish	2.048	0.1568	0.2131	0.7711	0.9102	0.0466
William I	2.305	0.6163	0.3161	0.5871	1.0101	0.1422
Grand naine	2.183	0.5892	0.2304	0.7241	0.8439	0.1466
Poyu	2.235	0.3794	0.2090	0.6402	0.8451	0.1008
GaintCavandish	1.927	0.3178	0.2484	0.9799	0.9464	0.0919
Butazu	2.560	0.8180	0.5197	0.3985	1.4072	0.1178

**Table 7:** Parameteric stability analysis of varieties for peel weight across postharvest environments.

Variety	Mean	Wricke ecovalence	Stability variance	Cultivar Superiority	Bi	SDi
Local Variety	15.61	309.6	14.44	322.06	0.3254	10.84
Dwarf Cavandish	33.70	198.7	111.14	25.99	1.2363	22.05
William I	37.07	275.8	136.50	13.37	1.3790	38.53
Grand Naine	33.19	91.3	46.21	34.69	0.7915	19.27
Poyu	29.82	85.5	39.31	58.75	0.7463	16.24
GiantCavandish	33.70	175.7	108.07	27.62	1.2128	42.78
Butazu	31.80	153.4	115.88	39.23	1.2993	25.12

**Table 8:** Parameteric stability analysis of varieties for fruit weight across postharvest environments.

Variety	Mean	Wricke ecovalence	Stability variance	Cultivar Superiority	Bi	SDi
Local Variety	63.57	544.6	69.5	1651.1	0.5102	79.4
Dwarf Cavandish	100.44	1171.1	354.0	249.7	1.2692	130.0
William I	114.69	604.3	306.5	45.1	1.2729	212.3
Grand Naine	102.14	472.3	160.0	197.9	0.8353	239.2
Poyu	94.23	770.2	81.3	397.7	0.4563	116.0
GiantCavandish	92.56	585.3	288.9	401.0	1.2427	151.9
Butazu	109.11	813.1	347.0	93.1	1.3761	163.4

## 4 Discussion

Based on principal component analysis; positive and high PCA 1 was obtained for peel weight, fruit weight and fruit length; however, it was negative and high for pulp peel ratio and pulp diameter (Fig. 2). Similarly a high negative PCA 1 score for pulp diameter and fruit diameter was reported [19]. The biplot of the PCA placed fruit length, fruit weight, peel weight, postharvest period, as well as Grand naine and Dwarf Cavendish near each other; whereas, peel diameter, pulp peel ratio, weight loss, as well Butazu and William I, plotted near each other (Fig. 2). Variety: Poyu, with small PCA1 and 2, were kept near the origin of the biplot. The local variety was placed separately from the other varieties, suggesting different postharvest characteristics.

Based on the cluster analysis, Grand Naine and Dwarf Cavendish; Poyu and Butazu grouped together whereas the local varieties clustered uniquely (Fig. 3). In line with this, six banana varieties were clustered using morphological characteristics and three cluster categories were depicted in the result [20]. The first cluster consisting of Kar-pooravalli (ABB), Njalipoovan (AB) and Yangambi (AAA); the second cluster was dominated by the group having ‘A’ genome with all the banana cultivars mentioned above with the exception of Karpooravalli (ABB) and the addition of two banana cultivars, namely Grand Naine (AAA) and Pisang Lilin (AA) and the last category of cluster consisted of banana cultivars viz. Grand Naine (AAA), Pisang Lilin (AA) and Nendran (AAB) .

Both multivariate analyses; principal component and cluster analysis results (Fig. 2, Fig. 3) indicated that production of introduced varieties can facilitate the development of the banana industry in Ethiopia; for these varieties have good postharvest characteristics, preferred in the domestic and international market. The local variety with a short postharvest period, might not be suitable for export market; however, because it can ripen fast without packaging at room temperature. Thus, the local variety may be restricted to local markets where there are limited fruit ripeners.

The possession of greater fruit weight, peel weight and longer postharvest periods by the introduced banana offers an opportunity for breeders to improve local elite varieties to be able to withstand the multiple biotic and abiotic stresses tormenting banana production. Banana varieties with high peel weight are often more suitable for international banana trade, as the peel helps the fruit to resist mechanical damage during transportation [5]. Accordingly, correlation analysis depicted that postharvest period was highly and positively correlated with peel weight, fruit weight and fruit length; however, it was negatively correlated with pulp peel ratio and pulp diameter (Table 1). Weight losses were significantly and positively correlated with fruit weight and fruit volume. The study points out that fruit weight and peel weight can aid selection to develop varieties having longer postharvest shelf life and quality with minimum weight loss.

**Fig. 1:**
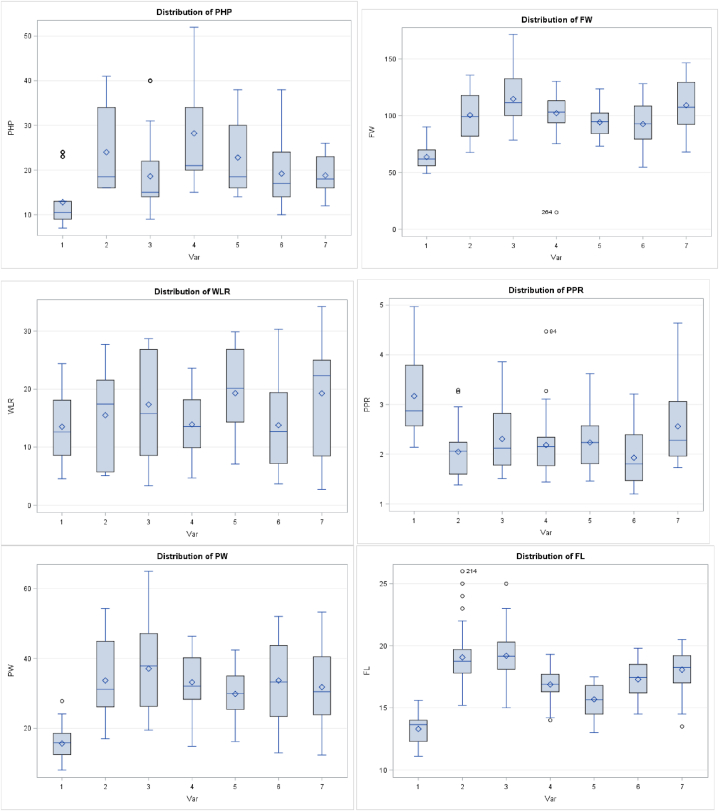
Q-Q plot of fruit quality traits of seven common banana cultivars grown in Ethiopia. 1 = Local Variety; 2 = Dwarf Cavandish; 3 = William I; 4 = Grand Naine; 5 = Poyu; 6 = GaintCavandish; 7 = Butazu; PHP = Postharvest life (days); FW =Fruit weight (g); WLR =Weight loss ratio(%); PD =Pulp diameter (cm); PPR =Pulp-peel ratio; PW = Peel weight (g); FL =Fruit length (cm).

The highly significant (p < 0.001) variation for the main effects of variety, temperature, packaging, and their second and third order interactions for all postharvest traits (Fig. 1), resulting from the combined analysis of variance, indicate that genotypes, environments and their interaction were important in governing the expression of these traits across postharvest environments. The presence of variation between packing at different temperatures, revealed the presence of dissimilarities between the various postharvest environments. The effect of postharvest environments was greater than genotypes and genotype by environment interaction, implying that optimization and development of postharvest environments and technologies for fruits, including banana, can reduces losses and improves quality in the market chain. Although fruit ripening trait values were not consistent across varied postharvest environments, preferable fruit quality was obtained at 20 and 25 °C with packaging. The genotypic effect depicted clear variation among banana varieties; banana fruits undergo metabolic process during ripening in the postharvest period. Metabolic processes are genetically controlled and affected by the postharvest environments [21]. Therefore, identification of better genotypes is crucial for reducing postharvest losses and improving ripening quality. The significant variety and temperature, variety and packaging, variety packing and temperature interactions (Table 2), indicated the differential varietal performance across the varied postharvest conditions.

**Fig. 2:**
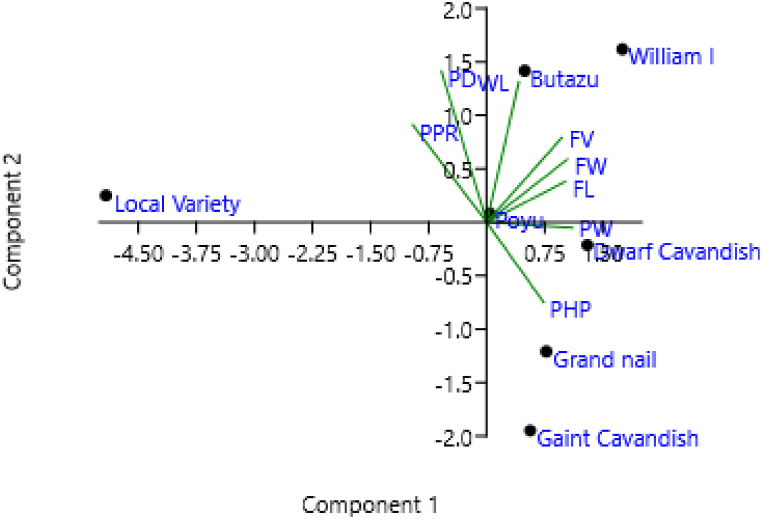
Biplot of the principal component analysis of eight fruit ripening traits and seven banana varieties. PHP = Postharvest life; WLR = Weight loss ratio; FL = Fruit length; PD = Pulp diameter; PPR = Pulp-peel ratio; FV = Fruit volume; FW = Fruit weight; PL =Peel weight.

**Fig. 3:**
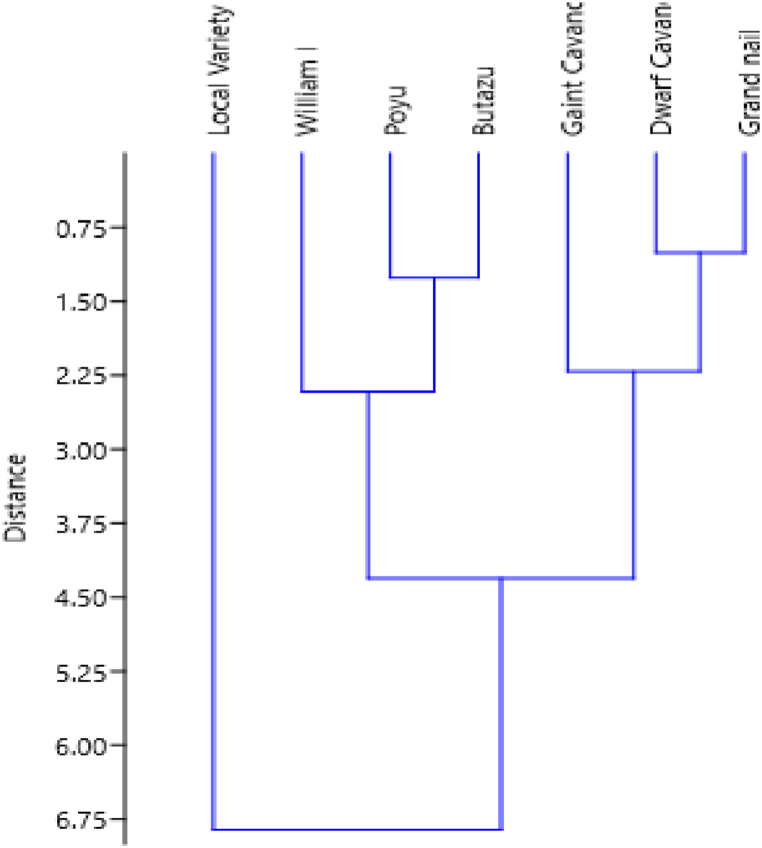
Cluster diagram of seven banana varieties used in the analysis of stability of fruit ripening traits of banana varieties across postharvest environments.

**Fig. 4:**
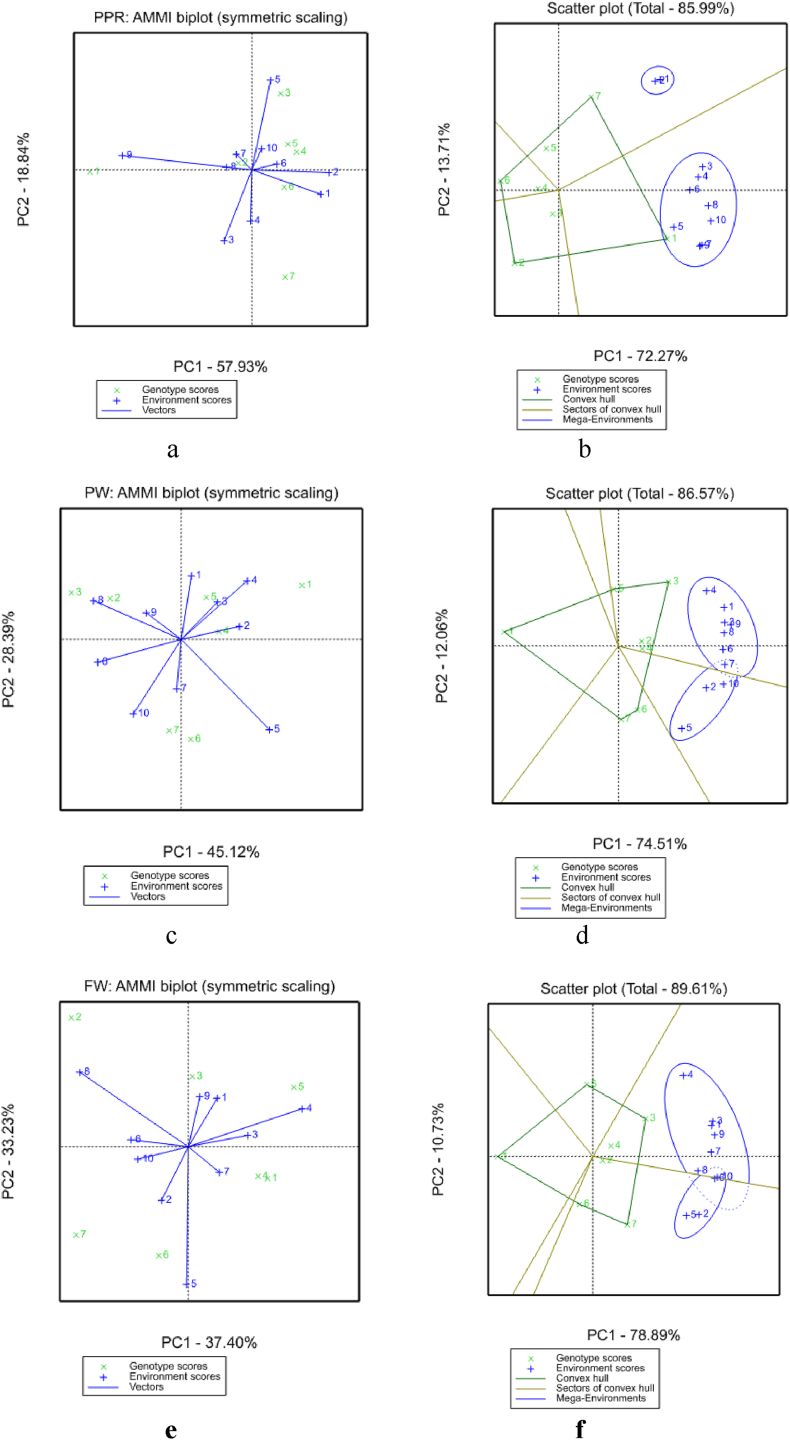
AMMI (a c, e) and GGE (b, d, f) biplot of fruit ripening traits across postharvest environments ×1 = Local Variety; ×2 = Dwarf Cavandish; ×3 = William I; ×4 = Grand Naine; x5- Poyu; ×6 = GiantCavandish; ×7 = Butazu; +1- 15^o^Cwithout packing; +2- 20^o^Cwithout packing; +3–25 °C without packing; +4–30 °C without packing; +5- room temperature without packing; +6–15 °C with packing; +7–20 °C with packing; +8–25 °C with packing; +9–30 °C with packing; +10- room temperature with packing.

Stability analyses helps to identify varieties with low genotype by environment interactions; and a lot of researches have been done and published on stability of pre-harvest traits, but research and publication on postharvest traits under postharvest environments is limited. In the present study, different stability analysis models were applied to identify varieties with consistence fruit traits and optimum fruit ripening quality across diverse postharvest environments. All stability analysis models (Additive main effects and multiplicative interactions (AMMI), GGE Biplot Eberharts and Russell’s coefficient of regression (Bi), deviation from regression (s^2^di), Wricke’s ecovalence (Wi) and Shukula’s stability variance (σi2) showed similar results. Accordingly, Gran naine; William I; and Poyu had better stability in the studied postharvest environments. Thus, these varieties are preferred for long distance markets, where postharvest environmental conditions are highly variable.

## 5 Conclusions

Based on the findings of the present study, postharvest life was highly and positively associated with peel weight, fruit weight, and fruit length; however, it was negatively correlated with pulp peel ratio and pulp diameter. The study points out that fruit weight and peel weight can aid selection to develop varieties having longer postharvest life with minimum weight loss Moreover; fruit traits of banana varieties, including postharvest period, weight loss and ripening traits are greatly influenced by variety, postharvest environments and their interactions. The magnitude of postharvest environmental effect is greater than genotype and interaction effects. AMMI and GGE biplot analysis identified Grand Naine, Poyu and William I as consistent for ripening traits across varied postharvest environments. Thus, these varieties need to be grown for both domestic and international markets. Further research on stability of physiochemical traits in different postharvest environments, development and optimization of low cost postharvest technologies should also be conducted.

## Data availability statement

Data will be available on request.

## CRediT authorship contribution statement

**Muluken Bantayehu:** Writing – original draft, Data curation. **Melkamu Alemayehu:** Writing – review & editing, Conceptualization.

## Declaration of competing interest

The authors declare the following financial interests/personal relationships which may be considered as potential competing interests:

The authors declare the following financial interests/personal relationships which may be considered as potential competing interests: Muluken Bantayehu reports financial support and travel were provided by 10.13039/501100005872Bahir Dar University. Muluken Bantayehu reports a relationship with 10.13039/501100005872Bahir Dar University that includes: funding grants and travel reimbursement. If there are other authors, they declare that they have no known competing financial interests or personal relationships that could have appeared to influence the work reported in this paper.
